# Supplementary material for: Toxoplasma F-box protein 1 is required for daughter cell scaffold function during parasite replication
Source: PLoS Pathog. 2019 Jul 26;15(7):e1007946. doi: 10.1371/journal.ppat.1007946 (PMC6685633; doi:10.1371/journal.ppat.1007946)
Supplement: S1 Fig — F-box domain sequences were predicted as described in Methods. To facilitate visualization of relatedness, acidic residues are in blue, basic in dark red, small or prolines in red, and hydrophobic in green. Positions matching the consensus motif are highlighted in yellow (hydrophobic), gray (acidic), green (basic), or teal (small). F-box sequences from known structures and other validated studies, and functional and interactome studies in Schizosaccharomyces pombe and Saccharomyces cerevisiae, were aligned to characterize the known diversity of F-box domains. The family of consensus sequences was used to search the genomes of Toxoplasma and other apicomplexans. (A.) Representative F-box motif sequences derived from crystal structures of F-box protein/Skp1 complexes and other well-known F-box proteins. (B). F-box motif sequences from the yeasts S. pombe and S. cerevisiae. (C). Candidate F-box motif sequences identified in the type II ME49 Toxoplasma strain. (DOCX) [file ppat.1007946.s001.docx]

**Fig. S1. Known and predicted F-box proteins.**

**A. Representative F-box motif sequences**

Protein^a^ Identifier Length Start Sequence^b^ Other

domain^c^

HsSkp2 GI:21260543 L= 424 94 WDsL**P**D**E**LLLGIFscL-cLPELLKVsGV-cKRWYRLasD--EsLWqtL LRR°

PDB_2ASS hhhhhhhhhh hhhhhhhhh hhhhh bb^d^

MmFbw7 GI:44887884 L= 629 200 IsLL**P**K**E**LaLYVLsFL-EPKDLLqaaqt-cRYWRILaED--nLLWREK WD40

PDB_2OVP hhhhhhhhhh hhhhhhhhhh hhhhhhhhh

HsβTrCP1/Fbw1a gb|AAF04464.1 L= 569 149 pargldhiaenilsyl-dakslcaaelv-ckewyrvtsd--gmlwkkl WD40

PDB_1P22 hhhhhhhhhh hhhhhhhhhhh hhhhhhhhh hhhhh

MmFbs1/Fbx02 GI:51315949 L= 297 51 LaELPEPLLLRVLaEL-PatELVqacRLVcLRwKELVDG--aPLwLLK CRD

PDB_2E31 hhhhhhhhh hhhhhhhhhhh hhhhhhhh hhhhhh

AtTir1 GI:332646898 L= 594 5 alsf**p**e**e**vlehvfsfiqldkdrnsvslv-ckswyeierwcrrkvfign amn1

PDB_2P1N hhhhhhhhhh hhhhhhhh hhhhhhhhhhh bbbb

ScCdc4 GI:14318513 L= 779 275 ItsL**P**F**E**IsLKIFnYL-qFEDIInsLGV-sqnwnKIIRK-stsLwKKL WD40

PDB_1NEX hhhhhhhhhh hhhhhhhhh hhhhhhhhhh

ScCtf13 Ymr094w/ L= 478 9 PSFNP-VRFLEL--PIDIRKEVYFHLD@-YAFWLRYDCLV-------- LRR

PDB_6FE8 hhhhhh hhhhhhhhhhhh hhhhhhhhhh

ScRcy1 GI:6322257 L= 840 3 DLLKVPEIVTNIASYL-STVDYLSFQQVNKRVYAIINGKNDSKYWSLK

SpPof1p GI:5913946 L= 605 110 LSLL**P**V**E**ISFRILSFL-DARSLCQAAQV-SKHWKELADD--DVIWHRM WD40

SpPoplp GI:162312293 L= 775 301 LTGF**P**A**E**ITNLVLTHL-DAPSLCAVSQV-SHHWYKLVSS-NEELWKSL WD40

SpPof9p GI:19112561 L= 467 6 FLELSYDILLEISTYL-DYKDIVHLSET-CKSLSYVFDD--KTIWHRF

HsJHDM1b/Fbl10 GI:54112382 L=1336 1062 aHVMHR**E**VWMaVFsYL-sHqDLcVcMRV-cRtWnRWccD--KRLWtRI JHDM

DdFbxA [DDB_G0276887](http://dictybase.org/gene/DDB_G0276887) L=1247 634 FDNL**P**E**E**VVQIIFSNL-SAINIVNLSLV-CKRFKMATDS--PILWKNL WD40

DdFbxD DDB_G0292312 L= 530 114 ILNLPSTIYAQIFSEF-PVKEILKFSLV-CKEFNKAINH--KFLWKIK WD40

LpAnkB GI:53751810 L= 154 6 fsdl**p**e**e**tivntlsfl-kantlgriaqt-cqffnrland+krelwQNL ankyrin

F-box motif consensus sequence* #xxL***P****x****E***ILxxILs#L-xxxDLLxLsxV-CbxWxxLsxx--xxIWbxl

^a^ Hs, *Homo sapiens*; Mm, *Mus musculus*; At, *Arabidopsis thaliana*; Sc, *Saccharomyces cerevisiae*; Sp, *Schizosaccharomyces pombe*;

Dd, *D. discoideum*; Lp, *Legionella pneumoniae*

^b^ Acidic residues are in blue, basic in dark red, small in red, and hydrophobic in green. Positions matching the consensus motif are highlighted in yellow (hydrophobic), gray (acidic), green (basic), or teal (small). At one position, two alternatives are highlighted. Bolded residues correspond to the PxE motif,

^c^ predominant C-terminal domain type: LRR= leucine rich repeat, WD40= WD40 repeat, amn1= antagonist of mitotic network (LRR-like), CRD= carbohydrate recognition, JHDM= Lys-specific demethylase 2B

^d^ h= α-helical; b= β-strand

+ deleted sequence = khlelhqlrqqri

@=76 aa deleted

* #=hydrophobic side chain, s=small, b=basic, PxE motif is italicized.

**B. F-box motif sequences from the yeasts *S. pombe* and *S. cerevisiae***

Gene name Length Start Sequence^#^ domains

*S. pombe*

SpPof1p L= 605 110 LSLL**P**V**E**ISFRILSFL-DARSLCQAAQV-SKHWKELADD--DVIWHRM WD40

SpPof10p L= 662 30 VLNL**P**K**E**ILIIIFSFL-DPRSLLSAQCT-CKYWKKLLSD--DLSWRTA WD40

SpPofllp L= 506 73 TEVF**P**E**E**VSLRVFSYL-DQLDLCKCKLM-SKRWKRLLED--PGIWKAL WD40

SpPoplp L= 775 301 LTGF**P**A**E**ITNLVLTHL-DAPSLCAVSQV-SHHWYKLVSS-NEELWKSL WD40

SpPop2p L= 703 239 LSNLPFSIVQSILLNL-DIHSFLSCRLV-SPTWNRILDV-HTSYWKHM WD40

SpPof2p L= 463 1 MRV**P**N**E**VCFNILSYL-EADELRCKSTV-CTSWRNFII---PTLWEKV LRR

SpPof3p L= 577 136 FRIL**P**R**E**VLLCILQQL-NFKSIVQCMQV-CKHWRDCIKK-EPSLFCCL TPR/LRR

SpPof7p L= 361 112 ILKL**P**D**E**VLLVILENC+DLRYLSSIALT-CKHFAKALR--ADSLYRSF

SpPof5p L= 348 1 MQSF**P**P**E**IWHHIFDHL-ISFDKFEAKNF-GGLLRICRSSYVGGLHAIY

SpApb1A10(Pof15) L= 243 31 STLL**P**V**E**VIDSVMQYL-PAHDVIQSSFA-SYPLTLIAN----KIIRAR

SpFbhlp L= 878 11 FYRL**P**L**E**IIPLICRFL-SVQDIQSFIRV-FPSFQTILDSSNDLFWKKK

SpPof9p L= 467 6 FLELSYDILLEISTYL-DYKDIVHLSET-CKSLSYVFDD--KTIWHRF

SpPof8p L= 402 63 IEPLNPDFLSAVDSIL-EIYFHRERQKE-KVHLAFLIQQ--DDFWKGI

SpPof12p L= 440 11 ASIFSHETLLHVLNDL-SAHDLAALERV-SRSWNSIVRR--SSVWHNL

SpPof13p L= 396 44 LFLLNRDIWSLIINYL-DAFDILRLMHS-SRQFYYWLRK--SAVDECC

SpPof6p L= 872 33 FGcLTINIYLKIFTLI-STPDLCNCRLV-CRKFQQLCDY--NSIYVKK

Residues deleted for the alignment: +=IRDLH

*S. cerevisiae*

ScYil046w=Met30 L= 640 184 ISIL**P**Q**E**LSLKILSYL-DCQSLCNATRV-CRKWQKLADD--DRVWYHM WD40

ScYfl009c=Cdc4 L= 779 275 ITSL**P**F**E**ISLKIFNYL-QFEDIINSLGV-SQNWNKIIRK-STSLWKKL WD40

ScYjr090c=Grr1 L=1151 317 LNML**P**S**E**ILHLILDKLNQKYDIVKFLTV-SKLWAEIIVK--ILYYRPH LRR

ScYdr306c L= 478 112 KMVL**P**W**E**IQHRIIHYL+TTGINMNYLLV-CRNWYAMCLP--KLYYAPA LRR

ScYor080w=Dia2 L= 732 207 VGNL**P**I**E**ILPIIFQRF-TTKELVTLSLV-CNKWRDKILY-HLDCFQEF TPR/LRR

ScYbr158w=Amn1 L= 549 166 Avfeipeiveniikmi\kqgalfscmmv-nrlwlnvtrp---flfksl LRR

ScYmr094w=Ctf13 L= 478 9 PSFN**P**-VRFLEL--PIDIRKEVYFHLD@-YAFWLRYDCLV-------- LRR

ScYlr352w L= 807 15 gaai**p**p**e**ivyqiltyq|lttfvksnltv-nktfshicqv---liyryc

ScYbr203w=Cos111  L= 924  187 INDL**P**V**E**IIAKILSEF?DQKTLVRCLYV-SKKFYKATKI---VLYRLP

ScYlr224w L= 369 8 LMDL**P**L**E**IHLSLLEYV--PNELRAVNKY$SLAWIAEDN----YIWAVV

ScYml088w=Ufo1 L= 668 8 LQDL**P**P**E**ILINIFSHL-DEKDLFTLQEL-STHFRNLIHD--EELWKNL

ScYjl149w=Das1 L= 663 49 LTKL**P**D**E**LMQEVFSHL-PQPDRLQLCLV-NKRLNKIATK---LLYRRI

ScYdr131c L= 556 2 FDKL**P**Y**E**IFKQIAWRI-PQEDKISLTYV-CKRSYESIIP---FIYQNL

ScYlr368w=Mdm30 L= 598 16 IDHL**P**P**E**IWLCISKLV-GTSDLHNLCLI-NRRLYLTITS--DEIWKRR

ScYbr280c=SAF1 L= 637 12 DAGLSPDIVQATLPFL-SSDDIKNLSQT-NKYYNTLLDFD/KILWHEL

ScYdr219c=Mfb1 L= 465 17 LTNLPLNLLFRILSHL-DMNDLQNIGKT-CTLLRMLANE--NIVYRNA

ScYnl311c=Skp2 L= 763 57 LMCLPTKVLLLILRTL-DFNTLVTLCQV-NSRFYNLITN--EFLFQNV

ScYmr258c L= 553 1 MAFQDQDIFIVFSHAS$NQNDLLSLSLT-SKKMHDMIAI--PRLYSNI

ScYlr097c=Hrt3 L= 344 100 LEILPDDILLRIIKKV^SGESWVNLSMT-CSTFSKLCFH-DSVPFKTF

ScYjl204c=Rcy1 L= 840 3 DLLKVPEIVTNIASYL-STVDYLSFQQV-NKRVYAIINGK/SKYWSLK

ScYnl230c L= 379 22 VSNVPYHLLKRILQKV-KIPQLLKLEK--SNVLLIFDDD---ELWLEF

^#^ Acidic residues are in blue, basic in dark red, small in red, and hydrophobic in green. Positions matching the consensus motif (panel A) are highlighted in yellow (hydrophobic), gray (acidic), green (basic), or teal (small). Bolded residues correspond to the PxE motif.

Residues skipped for the alignment are as follows: /=HS; &=LFL; ^=ILM; +=DIPEKEEKLNKTANGKKT; ?=ELGR; $=FYVLHNHSYKEK; @=76 aa deleted; \=57 aa deleted; |=18 aa deleted

**C. Candidate F-box motif sequences from *Toxoplasma gondii*** (type 1 strain ME49)

Name Gene ID* Length Start Sequence‡ domains

a.a.

FbxW1 TGME49_261370 1618 941 WTSV**P**A**E**VLSTLCQFL-AVEDLVAFQRL-DRRAYAVGSH--ATVWRAL WD40

FbxW2 TGME49_299230+ 636 16 PACLPTAIVFHVASFL-TPNDVCSMGAT-CRKWREVCCSDLQPLWREF WD40

FbxW3 TGME49_110910^ 3679 236 LESLSPCLLANLVSFL-LPLDIVSVSLC-SRSLLWLSRC--PFVWRRC WD40

FbxL1 TGME49_262530 979 173 FAEL**P**Q**E**VLELIFSRL-GLADLSRCLCV-AKSWHPPLNA---VFAKTI LRR

FbxL2 TGME49­­­_313200• 839 307 LSDL**P**E**E**LLQQILDCC-PKECLLVSHAL/RRRVLRLTPL---QCTEPR LRR

FbxO1 TGME49_310930• 808 444 FPFLDEPALSLLVPFL-FGRSLATCMTV-CPHWFMKINR---AMERMC

FbxO2 TGME49_215210+ 683 254 FNTC**P**A**E**CLQAVFHFL-HVEDILRMQVV-SSAFFSTIRDEIGAFTHIR

FbxO3 [TGME49_225900](http://toxodb.org/toxo/showRecord.do?name=GeneRecordClasses.GeneRecordClass&project_id=ToxoDB&source_id=TGME49_025900)+ 1461 160 LEALPAGCMYTLFAFF-DVSEVAELRLL-SRTVKAVVDS--PCSLRGC

FbxO4 TGME49_228380¶ 1726 42 LCFDYPLCFDYLLSFL-DLRDFLTLSLV-SHSLRDILLSDLTRAARCV

FbxO5 TGME49_243750+ 865 478 LCSL**P**Q**E**LLDVLPLYL-DAFALTRLSSC-CRLLHRLCGNRSDVCWRAK TPR

FbxO6 TGME49_258900¶ 577 3 DLLQHPDIVGRILSCL-AWRERRQLAAV-CVSWREAAET--SPCWSDL

FbxO7 TGME49_275780 978 211 IDALPDDLLCEMLLFL-PFDEVGASIPLVSRRFCRLALL--PYIWTFF

FbxO8 TGME49_305630 2045 61 SPDG**P**C**E**TIVFLLGFFLPVADLCSCSAV-CKAWWAVCTLQHQQLWRER

FbxO9 TGME49_359350¶ 1788 742 LRQLPPAILCAVLRFL-PCASVLAFGTT-CRYAHDLVQL--PAAWNLL

FbxO10 TGME49_215620¶•1930 280 GAEAPEDARGCLLAFL-TWNDLARLRAV-SRQLKRLVEN-AALSARAV

FbxO11 TGME49_203040^ 1894 311 FSDLPDVCVRICFSFL-SVEEILKYQFL-SRYIRRAIGL---DHVLPL

FbxO12 TGME49_278815¶ 2186 84 LHLLPSSALSLLLRFL-HLDDVCRLALS-SKQLYLHPDLNTPFAVAHL LeuZipper

FbxO13 TGME49_283890• 739 63 FAALDDAAFLAFLSTL\PLSALLSLSCA-SKFLLAALLD--EELWQSL JmjC

F-box motif consensus sequence^¢^  #xxL***P****x****E***##xx##s#L-x#xD##xLsx#-Cbxwxx#sxx--xx#wbx#

*Sequence identifiers are from [www.toxoDB.org/](http://www.toxoDB.org/).

^‡^ Acidic residues are in blue, basic in dark red, small in red, and hydrophobic in green. Positions matching the consensus motif are highlighted in yellow (hydrophobic), gray (acidic), green (basic), or teal (small). Bolded residues correspond to the PxE motif.

+= reported also in ref. 65.

^¶^= Identified by hidden markov model

• supported by interactome studies

/=AAAVRR; \=AEFV

^¢^ Consensus for this set: #=hydrophobic side chain, s=small, b=basic, PxE motif is bold italicized.
