# Supplementary figures and images for: Toxoplasma F-box protein 1 is required for daughter cell scaffold function during parasite replication
Source: PLoS Pathog. 2019 Jul 26;15(7):e1007946. doi: 10.1371/journal.ppat.1007946 (PMC6685633; doi:10.1371/journal.ppat.1007946)

## Slide 1
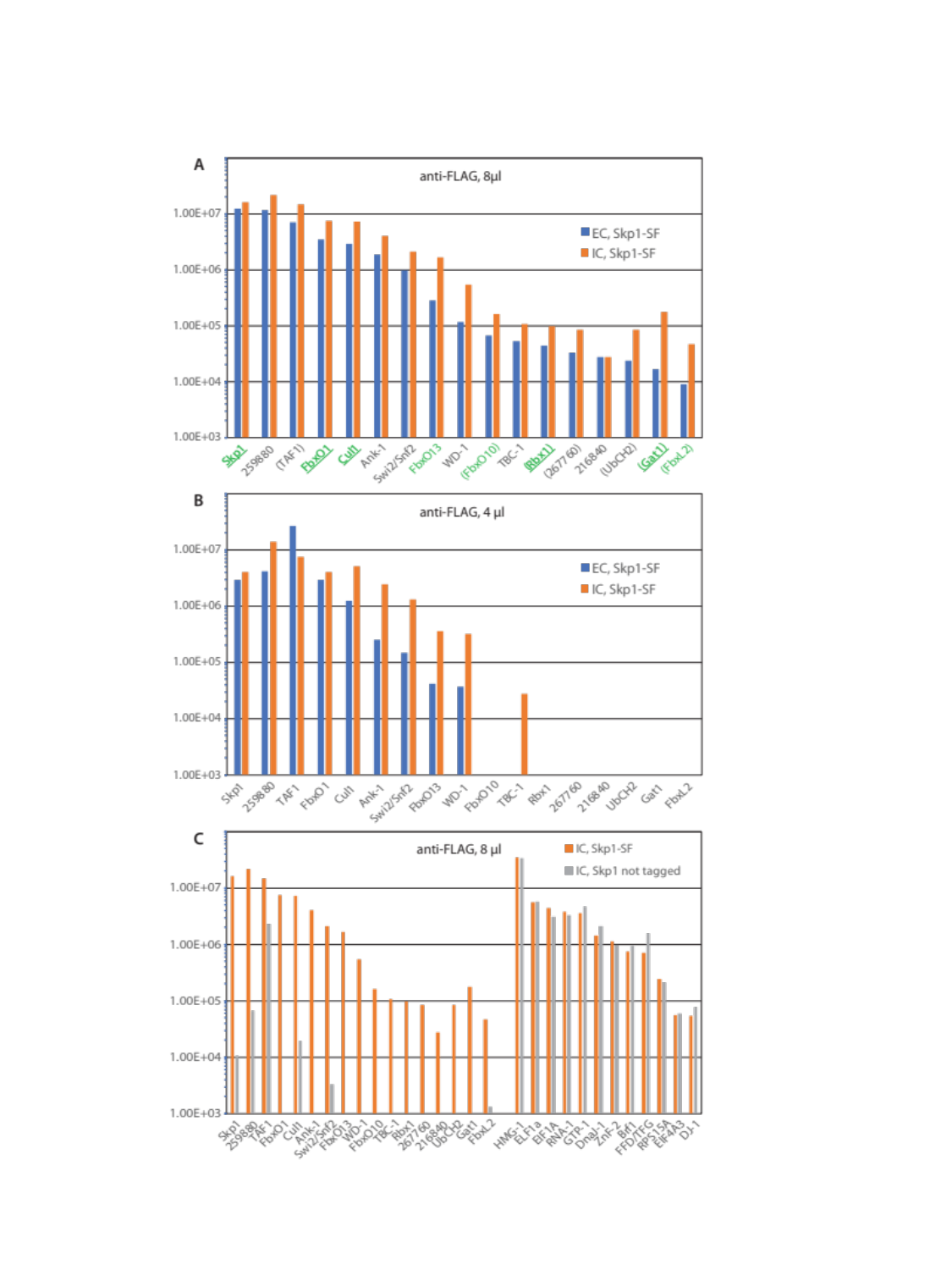

## Slide 2
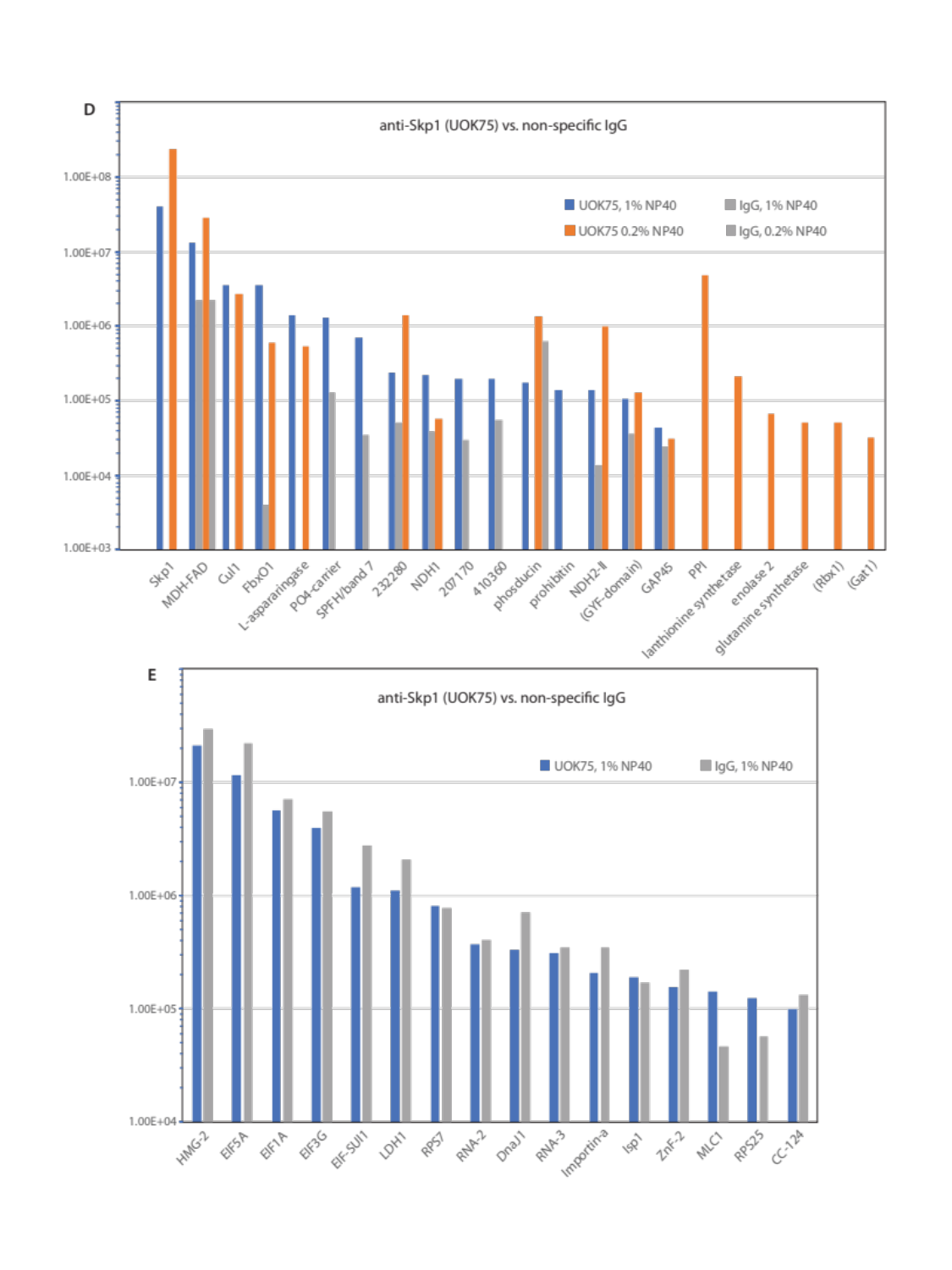

Supplement: S2 Fig — (A-C) Intracellular (IC) or extracellular (EC) forms of RHΔhxgprtΔKu80 (RHΔΔ) or TgSKP1SF tachyzoites were lysed in 0.5% NP-40 and the supernatants were incubated with magnetic anti-FLAG (M2)-beads. The captured material was eluted with 133 mM TEA, reduced and alkylated, trypsinized, and analyzed by nLC-MS2 in an Orbitrap mass spectrometer. (A) Quantitation based on abundance values calculated by the ‘area under the peak’ algorithm in Proteome Discoverer 2.2 from 8 μl sample injections. All proteins that were identified based on 2 or more peptides at 1% FDR, and were not predicted to reside within organelles, and were enriched ≥8-fold in TgSKP1SF extracts relative to RHΔΔ extracts, are shown. Additional proteins that were seen when criteria were reduced to one peptide and the protein FDR increased to 5%, are included if they were predicted to be in the TgSKP1 interactome based on F-box sequence motifs or other information. These are labeled in green, and in parentheses if seen only with the relaxed criteria. Proteins whose interaction were supported by detection with a different antibody in panel D are underlined in bold. (B) Same as in panel A, from a 4 μl injection. Assignments with higher confidence and at greater abundance were confirmed. (C) Comparison of protein abundance, as detected in panel A (8 μl injections), in SF-tagged and untagged extracts. At the right are data from arbitrarily selected examples that were present in both samples, indicating non-specific interactions. The average total Abundance was calculated for this protein set for each sample, and used to generate a normalization factor relative to the TgSKP1SF IC values, which was applied to each set of values in Panels A-C. (D, E) Similar analysis in which RHΔΔ tachyzoites were lysed in 1% or 0.2% NP-40 and incubated with affinity purified anti-Skp1 (UOK75) or non-specific IgG on magnetic beads. (D) Abundance values are reported for all 4 conditions for proteins whose enrichment in UOK75 relativ [file ppat.1007946.s002.pptx]

## Slide 1
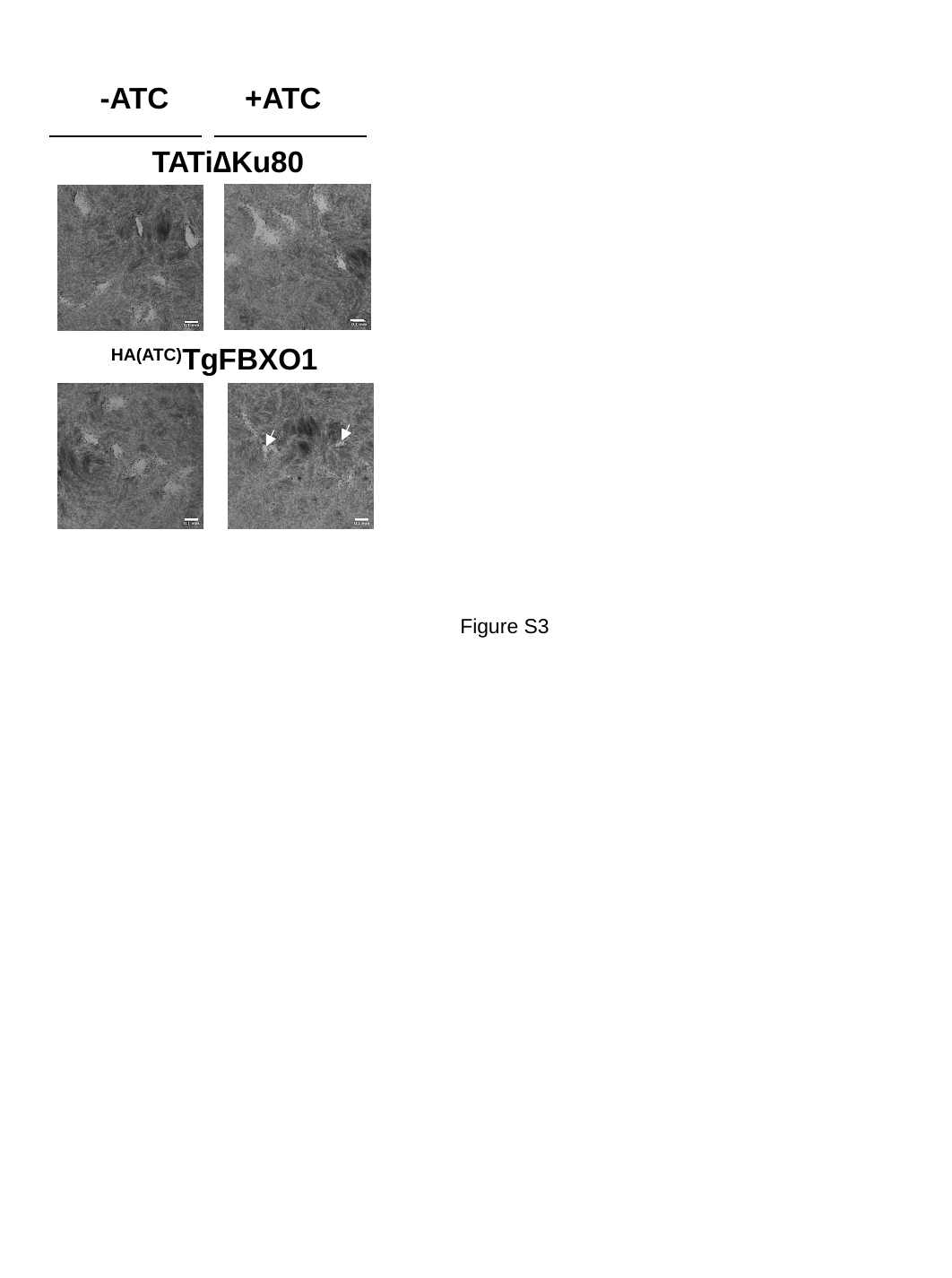

-ATC
+ATC
TATi∆Ku80
HA(ATC)TgFBXO1
Figure S3

Supplement: S3 Fig — Shown are lower magnification images of representative plaques from Fig 2D. (PPTX) [file ppat.1007946.s003.pptx]

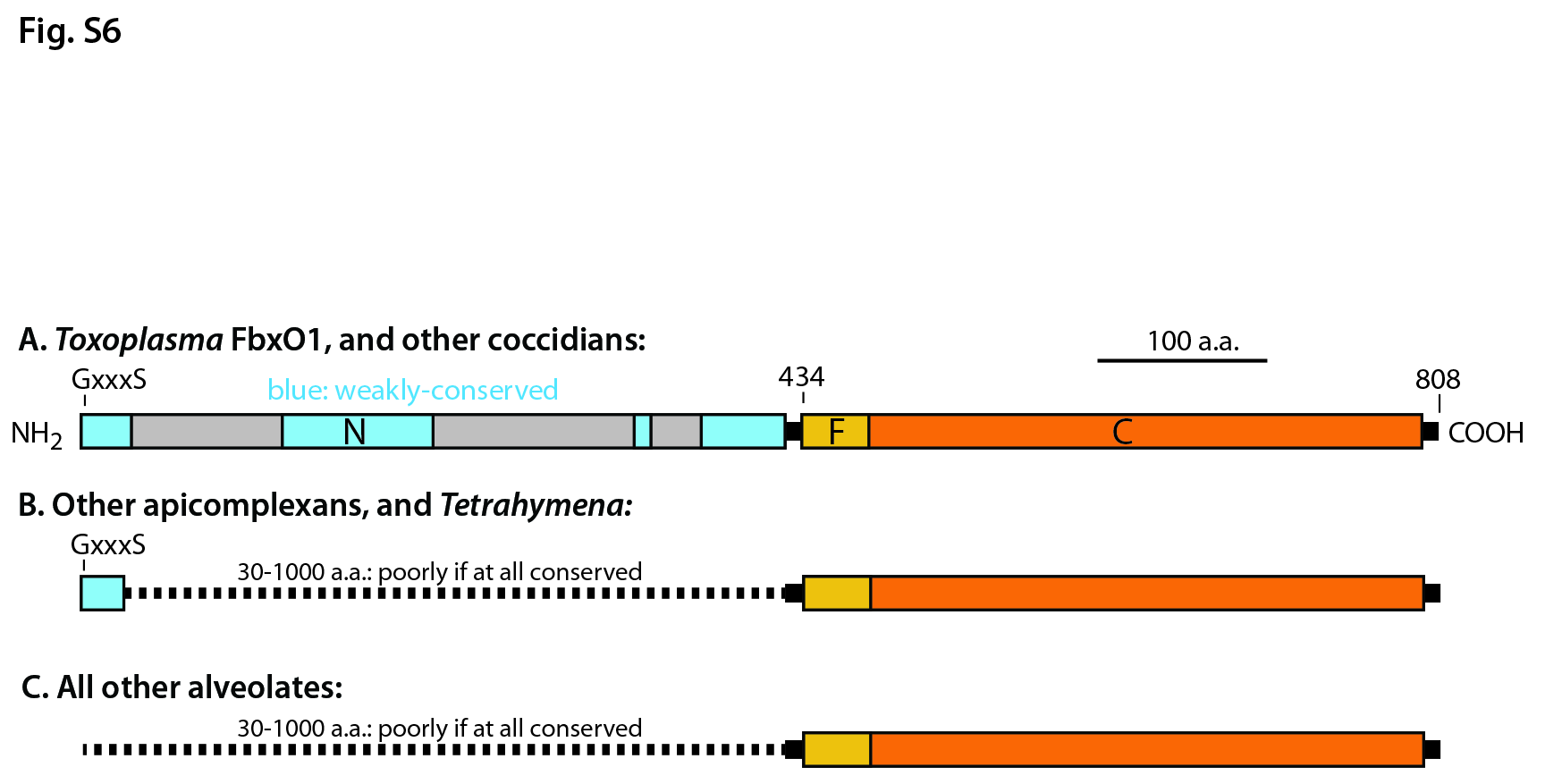

Supplement: S6 Fig — Sequence-based homology (S2 Fig) suggests that TgFBXO1 consists of at least 3 domains. (A). The three domains are conserved in FBXO1 homologues in Coccidians. The N-domain is conserved in length and general amino acid composition, but only the regions shaded in blue, including the N-terminal N-myristylation motif (GxxxS), are conserved in sequence. (B). In non-coccidian apicomplexans and other alveolates, the ~370 C-terminal region is generally conserved. This region includes an N-terminally positioned predicted F-box domain (F) and the C-domain. (C). Some alveolate sequences consist of only F- and C-domains, whereas others contain N-terminal extensions of variable length (dashed line) that bear no detectable sequence similarity to the N-domain of TgFBXO1. (TIF) [file ppat.1007946.s006.tif]
