# Supplementary material for: Toxoplasma F-box protein 1 is required for daughter cell scaffold function during parasite replication
Source: PLoS Pathog. 2019 Jul 26;15(7):e1007946. doi: 10.1371/journal.ppat.1007946 (PMC6685633; doi:10.1371/journal.ppat.1007946)
Supplement: S5 Fig — Sequences homologous to TgFBXO1 were aligned in COBALT and subject to manual refinement. To facilitate visualization of relatedness, acidic residues are in blue, basic in dark red, small or prolines in red, and hydrophobic in green. Positions of similarity in one group are highlighted in yellow (hydrophobic), gray (acidic), green (basic), or teal (small). Underlines separate phylogenetic groups: the top two groups consist of different coccidian subclades, the next two groups represent other apicomplexans, the next group contains a chromerid (apicomplexan predecessor), and the bottom group contains non-apicomplexan alveolates including ciliates and dinoflagellates. (DOCX) [file ppat.1007946.s005.docx]

**Figure S2. Homology of alveolate sequences related to *T. gondii* FbxO1**

**Code Accession Species Clade**

[Tg XP_002364337.1](https://www.ncbi.nlm.nih.gov/protein/XP_002364337.1?report=genbank&log$=prottop&blast_rank=1&RID=0BN63AAB211) TGME49_310930 [Toxoplasma gondii ME49] Coccidian A Apicomplexa

[Hh XP_008885908.1](https://www.ncbi.nlm.nih.gov/protein/XP_008885908.1?report=genbank&log$=prottop&blast_rank=2&RID=0BN63AAB211) HHA_310930 [Hammondia hammondi] Coccidian A Apicomplexa

[Nc XP_003885097.1](https://www.ncbi.nlm.nih.gov/protein/XP_003885097.1?report=genbank&log$=prottop&blast_rank=3&RID=0BN63AAB211) [Neospora caninum Liverpool] Coccidian A Apicomplexa

[Beb PFH34091.1](https://www.ncbi.nlm.nih.gov/protein/PFH34091.1?report=genbank&log$=prottop&blast_rank=4&RID=0BN63AAB211) hypothetical protein BESB_072430 [Besnoitia besnoiti] Coccidian A Apicomplexa

[Cs PHJ16413.1](https://www.ncbi.nlm.nih.gov/protein/PHJ16413.1?report=genbank&log$=prottop&blast_rank=5&RID=0BN63AAB211) hypothetical protein CSUI_009775 [Cystoisospora suis] Coccidian A Apicomplexa

[Em XP_013337306.1](https://www.ncbi.nlm.nih.gov/protein/XP_013337306.1?report=genbank&log$=prottop&blast_rank=6&RID=0BN63AAB211) oxidoreductase, putative [Eimeria maxima] Coccidian B Apicomplexa

[Cc XP_026193228.1](https://www.ncbi.nlm.nih.gov/protein/XP_026193228.1?report=genbank&log$=prottop&blast_rank=7&RID=0BN63AAB211) LOC34623623 [Cyclospora cayetanensis] Coccidian B Apicomplexa

[To XP_009689732.1](https://www.ncbi.nlm.nih.gov/protein/XP_009689732.1?report=genbank&log$=prottop&blast_rank=11&RID=0BN63AAB211) TOT_010000887 [Theileria orientalis strain ShIntoKu] Piroplasmid Apicomplexa

[Bab XP_012766453.1](https://www.ncbi.nlm.nih.gov/protein/XP_012766453.1?report=genbank&log$=prottop&blast_rank=13&RID=0BN63AAB211) hypothetical protein, conserved [Babesia bigemina] Piroplasmid Apicomplexa

[Pr CRH00939.1](https://www.ncbi.nlm.nih.gov/protein/CRH00939.1?report=genbank&log$=prottop&blast_rank=15&RID=0BN63AAB211) conserved plasmodium protein, unknown function [Plasmodium relictum] Haemosporidian Apicomplexa

[Cm XP_002140319.1](https://www.ncbi.nlm.nih.gov/protein/XP_002140319.1?report=genbank&log$=prottop&blast_rank=9&RID=0BN63AAB211) hypothetical protein [Cryptosporidium muris RN66] Conoid Apicomplexa

[Vb CEM03254.1](https://www.ncbi.nlm.nih.gov/protein/CEM03254.1?report=genbank&log$=prottop&blast_rank=10&RID=0BN63AAB211) unnamed protein product [Vitrella brassicaformis CCMP3155] Chromerid

[Im XP_004034674.1](https://www.ncbi.nlm.nih.gov/protein/XP_004034674.1?report=genbank&log$=prottop&blast_rank=17&RID=0BN63AAB211) hypothetical protein IMG5_116140 [Ichthyophthirius multifiliis] Ciliophora

[Pt XP_001454827.1](https://www.ncbi.nlm.nih.gov/protein/XP_001454827.1?report=genbank&log$=prottop&blast_rank=19&RID=0BN63AAB211) hypothetical protein [Paramecium tetraurelia strain D4-2] Ciliophora

[Tt XP_001028005.3](https://www.ncbi.nlm.nih.gov/protein/XP_001028005.3?report=genbank&log$=prottop&blast_rank=12&RID=0BN63AAB211) TTHERM_00498170 [Tetrahymena thermophila SB210] free living ciliate

[Sc OMJ83137.1](https://www.ncbi.nlm.nih.gov/protein/OMJ83137.1?report=genbank&log$=prottop&blast_rank=14&RID=0BN63AAB211) hypothetical protein Stecoe_15990 [Stentor coeruleus] heterotrophic ciliate (heterotrich)

[Pp KRX05854.1](https://www.ncbi.nlm.nih.gov/protein/KRX05854.1?report=genbank&log$=prottop&blast_rank=16&RID=0BN63AAB211) hypothetical protein Ppersa_03791 [Pseudocohnilembus persalinus] pathogenic ciliate

[Sl CDW85380.1](https://www.ncbi.nlm.nih.gov/protein/CDW85380.1?report=genbank&log$=prottop&blast_rank=18&RID=0BN63AAB211) UNKNOWN [Stylonychia lemnae] Ciliate

[Ot EJY65153.1](https://www.ncbi.nlm.nih.gov/protein/EJY65153.1?report=genbank&log$=prottop&blast_rank=20&RID=0BN63AAB211) hypothetical protein OXYTRI_14697 [Oxytricha trifallax] Ciliate

[Sm OLQ08676.1](https://www.ncbi.nlm.nih.gov/protein/OLQ08676.1?report=genbank&log$=prottop&blast_rank=8&RID=0BN63AAB211) hypothetical protein AK812_SMIcGEnE7829 [Symbiodinium microadriaticum] endosymbiotic dinoflagellate

*

Tg 1 MGNTESTAAELHDNYLAVLKSVEGRLGQYPSRTLSPRSQSSGVDRSRA-RVRPEEAYRDPGKRG-AGNRPRNLPSKGEK 77

Hh 1 MGNTESTAAELHDNYLAVLKSVENRLGQYPSRTLSLRSQSSGVDRSRA-RVGSEEAYRDPGKRD-AGNRPRNVPSKGDK 77

Nc 1 MGNAESTASDLHDNYLAVLKSVENRLGQFPNRSLSFHSQPESNGRSRV-RTNTEEACQELGKRD-AANRPRKLSSKGEK 77

Beb 1 MGNTESTPSDLHDNYLAVLKSIETRLGKSATSESSRRRASGAGGWSRAARKQSEETRPPSGSRERASSRPCKSGSKLDK 79

Cs 1 MGNTESAPPALHDNYLAVIASIEGRLGHLPARNSHAPTKSPR---------------RANGVCH-HSSGPAAAVLPRDG 63

Em 1194 MGNHPSHPSIPLDGYLAVRASIEARF-------------------PPSQELRQIKPTEATACWGAPGGELHATLSGGRP 1253

Cc 1 MGNRPSYSSVPLDGYLAARASIEARF-------------------SHAAASRQ-RTASPSGCCGT-SEDTFPTMSAMEH 58

To

Bab

Pr 1 MGNKISECKHRQNILYKNIKNIIKQRNDID------------------------------------------------- 31

Cm 1 MGSVTSAPSHDKHVYPKNNNSIEPLSRSYS------------------------------------------------- 31

Vb 1 MGNVSSGSSRFDARKEDRKFKIRCSIIKRL------------------------------------------------- 31

Im

Pt

Tt 1 MGIDISKPNQTQGTNKNSNLQFNNHKSETI------------------------------------------------- 31

Sc

Pp

Sl

Ot 1 MYSNFSKEQQFQQQQQNNFQQQQFLQQHHTRI 32

Sm 20 VGNLRRQLQQRALRFPEELCVGKTLKLELLQQVGSEVVVCSHEESYRVAFEAGQLALTVSPESGDQDDAASDERFEMDG 103

Tg 78 PSR-----SAH-KSG-STSSVDQFNNG-PGANRKHGKDSVQRRDDAPP-QADQEHSSLQPA----------DGKRRPAAK 138

Hh 78 PSR-----SAH-KSG-STSSVDQLNDG-TGANRKRGKDRVQRRDDAPP-QAAEEHSSLHPA----------DGKRRPAAK 138

Nc 78 PSR-----SVQ-QSG-LNSSVDHSSAS-ADA-KKRGKERAHRRRDAA--QSDEDGNSSHPS----------DGKRR-TAK 135

Beb 80 SAS-----ALSSKSF-ADGNQRCSSES-LGS-KKRGKELAHRRQDTLP-HSDDEKKSSAKST---EGRRGHDAKRQ-TGS 146

Cs 64 PKC-----PVPDNSE-ISGTQSPGTGG-LDS---TGTSESSPRRNSGT-IVPQERRGKPPLT-------GDPGQVEGLGE 125

Em 1254 QEN-----PVA-GEP-CRSNVPLLLNR------------------------VAAGALVPPTL------------------ 1284

Cc 59 HPC-----SPH-REH-AKFSREMCLEQ-RGA--------------------VSSGLVLPPSL------------------ 92

To

Bab

Pr --------------------------------------------------------------------------------

Cm --------------------------------------------------------------------------------

Vb --------------------------------------------------------------------------------

Im

Pt

Tt --------------------------------------------------------------------------------

Sc

Pp

Sl

Ot 33 PSQNNNNSLGAGSMSQSIPNLRSFVEQRLNINQLEEDDLVSRGSETSNLKKPQFQKQGAKTLKTSDQNYKKNLFSAMTSR 112

Sm 104 LPMRRQDAAAGAKEYLEKHELLPYLQGLLETVCKEKPANPYRFLWRQLGLALDTSPPKKPAQNP----TGPTGKAQSARE 179

Tg 139 GETPNVESTATGSDWGDsAVNDF--AEEDSCAVESPAHFKL-qTKESGVKAPSPRLFPCETLYEVTSQIDLaDE 209

Hh 139 GETPNVESTATGSDWGDsAVNDF--AEEDSCAVESPAHFKL-qTKESGVKAPSPRLFPCETLYEVTSQIDLaDE 209

Nc 136 GEPPNLESTATGSDWEDaAVNDF--AEEDDCAVASPAHFKL-qTKDSGVKAPSPRLFPCETLSDGPSQRDLEDD 206

Beb 147 SASGVNESTAAGSDWEDsAVNDF--AEENECVVPSPVHFKL-ETKDSGVKGRSPRLFPCESELPSITDL---DD 214

Cs 126 GQGGDRDLAATESDWEDaTVNDF--AEEEVTSLSSSAHFKP-qSKQSSVKAPSPRLFPSTSALKLSSDSD--DG 199

Em 1285 ---SVAETTDTNSDWAPDEFEaA--ASSEILGGPSQSFTRE---KAPRVRAQSPQPFPSMS------------- 1337

Cc 93 ---SVAETTDTNSDGAPDEFEGA-qAAEDMFSRLSQSFTR---VKAPHVRARSPLPFPSMA------------- 146

To 1 MSNYTPTNRRIRCFSFRRRRNVFDYVNV 28

Bab 1 MANYRGFFLRFKGLCARRMDA--SLVRE 26

Pr 31 ----------------------------------------------------------------VKNNIYICES 40

Cm --------------------------------------------------------------------------

Vb --------------------------------------------------------------------------

Im

Pt

Tt --------------------------------------------------------------------------

Sc

Pp

Sl 1 MNTNPSIELFNNLDYQRPsKLINEMnDCLQKLDLDDSHNLEP-QSFEDLNGTIPCDFIPNSMTMSQACL--qST 71

Ot 116 QQQESQLNNSQLMMSQRsLtSLHQHDqSKLNQSQIYGGGQLNGLSANKNESQFLHLNTSQNINKSQFLQRsqSP 189

Sm 180 PEKSTRPGTPAELEGsRaTTPLPPPLRPTSEEAGTGCVVSA-DVLPEPLKSEIQEALEAPSESHLQWLHAVaGV 257

Tg 210 KTQVED[1]ELSSLPSSP TAS AEVATDKPGEGR[9]GRSAEGDRGP[9]AD AKEN-ESDNSHGESDQDL 287

Hh 210 KTHVED[1]ALSSVPSSP TAS AEVATEKPGEGR[9]GRSAEGGREP[9]AD AKEN-ESENASGESNQNL 287

Nc 207 EKTEVD[1]SLGSSPGSP TAS AGSVTGATG--- GRSSDARKER AE GKGK-DPVNTSDKSKRDP 263

Beb 215 EETQAG[3]ARESAPETP SAF AEGAEAQER--- ARKNSVSKKE AK[5]SRGD-KHARHSGEQKAES 278

Cs 200 RGEEAD[1]AFHTRDDER NGM ATEEPEKEKE--[6]AAQIDSSLSS[9]GN[5]VTGD-SSESKPNPDMVAK 277

Em 1338 ------ -----HCSS TVS RKAGTHKQQLPE -REISGSSSR --[1]SRSN-SSGSNDGGSACST 1383

Cc 147 ------ -----NCSP TVS KGSDAQQAPLCN -SSIAIINER --[1]YTLN-SERSPNAVGC--- 189

To 29 PTQATL[2]LHQPTNTQR E-- EPPEPSKHDDNR[5]----------[5]EP[7]-----YHPYVTNYHSTEK 90

Bab 27 PTVAVP[2]--EPRPVEQ T-- EVVEPSTTGDAS[9]PIENAQSPSS[9]EE[9]VTEDVYDPLIMNFSPKIS 111

Pr 41 NEKDLF[5]IFELYNTHE ENT[1]KKDNENKENEEN[9]KNEKEKKKNA[9]SE[9]KRDDEGKNVKEIDEQTKK 133

Cm ------ --------- --- ------------ ---------- -- ------------------

Vb ------ --------- --- ------------ ---------- -- ------------------

Im

Pt

Tt ------ --------- --- ------------ ---------- -- ------------------

Sc

Pp

Sl 72 PHLFR-[4]GLECTQSSP QIN[4]SQQIKAALFGGT[9]KIQQQNKDDD[9]LK[9]QVQSqFSTNYKQKNPYKS 165

Ot 190 NNIYQP[5]GNYASQFDP DLF[4]AMKKQYSQGVAQ[9]YYIQQNDDKI[9]QM[9]PLQEVIPQNQNQNGYAFK 285

Sm 258 EDFENV[5]ALFQSSIQQ[151]TAS[3]KVSAQEPPELAR[9]LTERILCRTA[9]EP[9]REQDaEDQGRGGKEDMPE 503

Tg 288 RRRSDGRGASSQRSR--------DEASNSGEPPSQSKPSQL SKQIRKKELAPEPLRAADIDLSSDVRVGASLKGGK 355

Hh 288 RRRSDARRASSRGSR--------DEASNSGGRPSQAKPSQL SKQIRKKELAPEPLRAADIDLSSDGG-GASLKGGK 354

Nc 264 RQRSNGSVASS-GTS---------GACLHTGQPHAARKSSP PSKQSRQKEQVTQLSAADIALPSHAS-GGCTQEGK 328

Beb 279 LEREQASGSER-GSP---QKKPsRGLSCSEARRSSAKNSAS LTNGRKKERQPQALCAEEVGLSSAAG-TGVRKKGS 349

Cs 278 LSETKVGRSLS------SKKStnSLAGTSTTRPSKSPAPGQ PRSRGPGRNVPEPLRAEELEYAVTTA-SVCSRRG[11] 356

Em 1384 RKVYAVSAFPGPSSR----PKQLDATLVASSGGAQRPRQDR SQQKNSSRSPPGACIADQHKAGFPVG---TRQTR[ 3]1454

Cc 190 ---------PQPKRK----PQQPAEALIAPTAG-------- -----------------------PHQTKQLGTEA 223

To 91 YAKKPTSKKVTPKSSIGEPPPPPKPPLNHAPSGPKYLE---[4]DLQTDFQKLSTETITR------------------ 148

Bab 112 RLRAEAADSDTCKSPtRDVNSIsQAAATSTSSGHTAEQGVF[4]---TLFSDVGSKPMAE------------------ 169

Pr 134 TNENHKKNFELDYGSFYNIEEKnNICKNNNNNKLLKKEKLL -KHIEVKNEKSQKLKNTEKTENVLLK-KISLNKN[ 9] 215

Cm 31 -----------------SNVELPSVTPVSRSQSIQTLSAPL[2]DSVSDLIDRISSRLNKNYRIVKYLAL--ACPKDY[ 8] 95

Vb 31 ----------------------------------------- -------------------GPPGAPMPETVPPPS[ 7] 52

Im

Pt

Tt 31 ----------------------------------------- ----------------SETIQKMILRVQSQIEKG[12] 56

Sc

Sl 166 RQIS---IHTNAYLAtDVG----DSTSHKQEIFSTAQTTQM[4]NNHQRVASNT--KSFFNQTNNNTFISYDNNMINE[ 8] 243

Ot 286 RATNDSVMLSNSFHNtqAPLPKVGQNQYQNQIQDFEDSQAQ[4]QQQLLSNYERAKNLDLSQ--QNKTHTLNSSGKK-[ 6] 367

Sm 504 AASQGQGGTSPEARAKRLMELRRSFSALKQSHGLLQRKVSK[5]DEVRRNTDQAVQLLRDAFVSQRSESD--SKEEKG[10] 591

Tg 356 GGKGDAEGDNVADLTAGFEGFSPYSPSYSLGFSQPSSKK--AAKTTAENSKAHLPG-GTRGSGNNTAVD---RDACIYGN 429

Hh 355 GVEGDAEGDTVADLAAGFEGFSPYSPSYSLGFSQPTSKK--AGKTTAESSEARLAG-GARGSGNNTAVD---RDSCIYGN 428

Nc 329 E-KGWADGD-LAEFAGDFEGFSPHSPSYSFGLTKLPKKA--IAKSGKTEERPPSAGTSARGSGNKASAD---ADACIYGN 401

Beb 350 K-ELTAEKG-GSAAAAQFEGFSPYSPSYAFDAPTPPIKA---TTTGRTEARASCRG-GGRGDGGSAGPEED-ADSYIYGQ 422

Cs 357 GASSTQEKR--PVSAVDLDSFSPYSPSYALGITTPTTNA--TTTTGSTEDRGSSRG-GFETEGDRLGS-----DRLIFGD 426

Em 1455 GAPEGRAAGVPSAGAEEHEMFCPLSPNSALGSCTPSK----ASSKSAPKTMGQTAV-FVGTTSSSHGMPA--ERNISYGP 1527

Cc 224 GTPSGRPSGLPKACINESDMLCPLSPNSALGSISPPAVAFKGASAKSTETLPETGA-PVQGTSKRF--------SVSYGP 291

To 149 ---SSRKGSVTY-----------------HSFTREDHAP--SRDVLGVGSRFLIGGKLSDGKPSMESRVWDMIERRGWG- 205

Bab 170 ---SAQKNHVS------------VLDDRIKTRTSPDFSP--S--------------KLAKCRSSL--RYYDLMGHR---- 212

Pr 216 DDESTFFNDENNNTILTKNSHMDNKELFKSTFKCIELKLLPDDDSYTGNIKSNIAEINYSNNIKIKENKNEINENLKNNK 295

Cm 96 EENTYVKKDEKFKWLNLFSHNLRYSGYDLQTGDKNSEILDtGNETGALNPSSKQHCLGSTCQFKGHSLYEDSKLYVVYGH 175

Vb 53 NLFGSAV--------------------SAAGASTPTS----------------SSGRGRSHSPRSSLPPPPPSPSAYYGH 96

Im

Pt 1 --------------------------------------------------------MQRKESKKATIPKMMARVLQQQEA 24

Tt 57 SMQGRQQIQQQQQQTQNQYYQTPQGQMQMQQQQRFQNMQYQPGNYQQVNGRIIQMNGQNQQPQYGYQNYQQLKIKVNCNV 137

Sc 1 MGITNTKPKDSEEVITSREYRSELIQDQRKRRHKTFAcMVQRVLI 45

Pp 1 MTK 3

Sl 244 -STNPQNQNNQIKQQIQQEKPRKKDKKPNNDDEFVNNYK----LALQQYQEDMNKQAYSHKKRLVSYDQDLKLRQNLRRK 318

Ot 368 ESHQILPSNTVPDSSQN--QNSNLSNVKERDSMLPPYKI-----KIKGNSNMKQEQLLQLTNRKSSSNLYTNYQHPIQRV 440

Sm 592 RIRKHPASGASLVEAMGGTSSQPIDVRVTDRKSLTKELCLRRKDYESMQHRYCVEDLTNSQENSRSRMAHLLHRAAIRDP 671

. Predicted F-box domain .

Tg 430 -----KKNHFPFLDEPALSLLVPFLFGRSLATCMTVCPHWFMKINRAMERMCGP-ATKG**F**QQMYSKYLEVWGSA-- 497

Hh 429 -----KKNHFPFLDEPALSLLVPFLFGRILATCMAVCPHWFMKINRAMERMCAP-ATKG**F**QQMYSKYLQVWGSA-- 496

Nc 402 -----KKNHFPFLDEPALSLLVPFLFGKSLATCMAVCPHWFMKVNRAMEHKCAP-VTKG**F**QQMYSKYLKVWGSA-- 469

Beb 423 -----ERNHFPFLDEPALSLLVPFLFGKSLAACMAVCPHWFMRINRAMERMCAD-VTDG**F**AKMYANYLKLWGGA-- 490

Cs 427 -----EGNHFPFLEEPALSLWVTFLLGKSLAACMAVCPHWFMKIDKAVSRMCAP-ITRD**F**AQAYSSHLEVWGSA-- 494

Em 1528 -----ACNHLPFLEGRALQALLPFLFGRTLGICMSVCVHWFMRISNGLAEMCSP-IVRG**F**EASYGKYLYPVNAT-- 1595

Cc 292 -----LCNHSPFMEGRAVQILLQLLLGPSLGACMGVCVHWFMKISDALADMCAH-LSKDLEASYGKFLSPVSAT-- 359

To 206 ----MVHENWNMVGARAFNSIVKYLTGMEISLCRQVCKGWYQNVTDVMVMRSEV-IVSA**F**KKAYKEKLEYDRSY-- 274

Bab 213 -----ISEDWADLNSRVYDALLQYFTGSDLTAGQKISKQWSDNVRLLMGKKTAK-IVAA**F**KVAYQGQLDFESSY-- 280

Pr 296 ---ILnKFLNLLKEKKILQNILSFLNCRDLLSFQKTCSIIYIYVSDFLDYICLN-IYSN**F**KRIYDFYFTPFNFF-- 365

Cm 176 -----LNNHKPFLSPGIIDTFLPFILDETLLTCFTVCPHWFLTLVCHMNQVYSHLIDDQ**F**KENYKGYLELEHAT-- 244

Vb 97 -----RANHMPFLEPAVMRRLIPFLWGQSFVRCTAVCPQWYMMLLAGVDDILQE-VDGK**F**DRRFAPYLKMEHTR-- 164

Im 1 MKLPFLAQNKIFFFLF-DEQINIKLINSLFYYKFSEVLEFEMVN-LDNK**F**IKNTMSFLSFKDS--- 61

Tt 138 -NCNVNEYLFQKLPSSVLYMILSFLM-NDYISIIMVSPLWYYKLHECIETELLK-IDND**F**IKSYSNALLFKNS--- 203

Pt 25 TDVLTRFLFIKQGQDRIRRNILGFLI-ADFTQLILVSGQWYVGFHQTLKEWLED-LDNR**F**IKAHLHILSFKNSDFL 98

Sc 46 ---TPSKTLIDTIPNDIFFIILQFLT-PEIPTLLAVSPKLHVKVYETIDTAFNA-IETQ**F**AIIHSNLLCFKKSF-- 114

Pp 4 QLEYQKEKIYNKDKNDVINKMIQRVQ-INLEKNKENTRPSNNAVNNQMESFRVPSMDSV**F**NNDNTNLQNKMTT[66] 149

Sl 319 --IKSVVQDPAQVPMNIFYTIISYDG-KLFKKFLCLSNGMKNHILFYVFDKTKK-ISHD**F**HRKYGQLLELQSRS-- 388

Ot 441 ---IQFGGNNCPIDTEIVMMILLFDM-PNFRKYLCLTPQWHHSILEAMDEYFKK-VECN**F**VMKNYEYLMFKKS--- 508

Sm 672 EIcKGGENSQIFMEAGMLPLVISFLFGGALIRCFEVCPLWFVVYYKTLDQVFSK-IDKG**F**KQYYGQSLHLEYAR-- 744

Tg 498 -VKLQPLQTVGDGGV**R**V**D**WVIFAKVLPECE--GHILDISYTYSYIS[43]LSPCGQP 590

Hh 497 -VKLQPLQTAGDGGV**R**V**D**WVIFAKVLPECE--GHILDISYTYSYIS[43]LSPCGQP 589

Nc 470 -VKLQPLQTAGDGGV**R**V**D**WVIFAKVLPACE--GNILDISYTYSYIP[42]LSPCGQP 561

Beb 491 -VKLQPLQTADDDGV**R**V**D**WVIFAKVLPRCA--GNILDIGYTYSYIP[44]LSPNGQP 584

Cs 495 -VKLQPLQISEDDGV**R**V**D**WIIFAKVLPRCA--GNVLEVGYTYSYLP[42]LSPSGPR 586

Em 1596 -VKLQALHTTEGNGV**R**L**D**WVITAKVLPPSA--NHVLSLGYCFKYRP[50]RPPSCAL 1695

Cc 360 -LKFLPLQIAGAKGT**R**L**D**WVITARVLPPAA--DNVLSLGYCFEYR-[47]LRPFSRT 455

To 275 -ILFQPVLTA-TKSL**R**I**D**VIIRAKIQPYCT--LHKNSFGYTYSYQS[49]------- 365

Bab 281 -MICQSVFTA-QKGM**R**I**D**LIIRARVLAWSV--NSKNTLQYTYSY--[ 5]------- 330

Pr 366 -YKYEYLYTD-KPSF**R**L**D**CILISKIKKGCI--GYNNRFGYKYNYIY ------- 409

Cm 245 -VTMQPVLTV-CGSV**R**I**D**RVLYAKVLKSCA--KKCVTIAYNFKYDK[29]------- 319

Vb 165 -VEWTQVHVA-DPGI**R**I**D**RIIVARVTEKCV--GRTITVGESFRLLP[111]------ 330

Im 62 YVSYKTQKFNNIKGI**R**M**D**RIINAELLPNIK--SNIIYLFYYIFYFL[19]------- 124

Pt 99 QTSFWVDSTKTKKLF**R**W**D**RTIISEVMSGFN--GKCITIAFKYNHK- ------- 141

Tt 204 YLTIKPIKVNNHFGF**R**M**D**RNICAEVLPSIS--NKTVSIKYTYKSFR[ 6]------- 253

Sc 115 -TDFTRMTVSDKKGI**R**I**D**RVIVAEILPYLN--GKTLKIRYNYRQAH[ 4]------- 161

Pp 150 HVSMAPIKISKNKGF**R**M**D**RNILAEILPTIK--NKCVTITYTYKIFQ[ 8]------- 201

Sl 389 -LIVKPLSFGKEEGV**R**L**D**LSLRVKIKKEAK------KIQGKSIIFS[13]------- 440

Ot 509 YTNSSLIHFCGQKGI**R**V**D**RIIVCEVLNSNKMLNKCLRISYSFKYKS[ 4]------- 558

Sm 745 -TDWSPVHVA-EAGI**R**I**D**RILIARVTSHHV--NHCTKVSYGYAYKS[13]------- 799

Tg 591 CPTNMPS-RVFTVS**Y**SLAAGAAKSSRTLWMHRDLCRF HG-DETGVAAMGSVSSVCVGDFVEVAVTVYNGGGR 660

Hh 590 CPTHTPS-RVFTVC**Y**SLAASAANSSRTLWMHRDMCRF HG-DETGVAAMGSVSSVCVGDFVEVAVTVYNGGGR 659

Nc 562 CPTHTPS-RSFTVS**Y**SLAASAAHSSRTLWMHRDMCRF HG-DETGVAAMGSVARVCVGDFVEVAVTVYSGGGR 631

Beb 585 CPANSSS-RSFTVS**Y**AFATSPANSSRTLWIHRDMCRF HG-DETGVAAMGSVARVCVGDFLEVAVSVFSGGGR 654

Cs 587 CPSHTPK-LTFTCS**Y**AFAVSPANGSRTLWIHRDMCRF HG-DETGVAVMGSIARVCAGDFVEVAVSIYSGGGR 656

Em 1696 GEATEH--RREAPM**F**SVAVAPAASKRRLWLHRDMCRF HG-DETGQAVSSHLGPVCVGDFVEVPVVLSNGIGA 1764

Cc 456 GESPKAGCRREVPT**F**SVAIAPAGSKRRVWLHRDMCRF HG-DETGQAALAPLGPVCVGDFIELPVVLANGIGI 526

To 366 --GKKSV---FISK**F**IFEVLKKNSCRTITFTRDLSSM HA-DDINLATSASVCQVCEGDYIEVPITLISAIGA 431

Bab 331 --ARQPT---YANR**F**VFECLRRNTRRTVSFMRDVSSV HG-DDLHVATAESVSQVCEGDSIEIPVVLMNALGN 396

Pr 410 ------RKSSYYAY**F**NFNVLKKNSSRIIEIHKDISYN NG-DDINVSHIIN-NYVCSNDYICIPINLYNFIGN 473

Cm 320 --KSSDINEAYLSI**Y**KFEALYTGTRRKQWAHKDISRC HS-EEVCVAQTTMIPSVNVGDRIEIAVNLSNSFGV 388

Vb 331 GPTSPPRIGPFEYR**Y**KFQTLPAGRSRQLWVHRDMCRF HG-DETRVATVQNVATVCVGDRVEIAVPLYSPRGC 401

Im 125 ---TRKKDQIYSCK**Y**QFDAIETNKNRIIWAFKEECKV[17]HNYDQQKQTFVQAITPIKVGDNIQIAINYYNLGGM 210

Pt 142 ----------YRSQ**Y**KFDVLPQNSKRVIWIAREQTK- HNFES--VTQVMNMQPILSGDCVKIAINFYNKMTF 200

Tt 254 ---SQQKQKKYLCE**Y**HIDAVQKKSSRTIWAYKDDCN- HNYDKKKVAFIQPITPVKIGDNIKISVNFFNMNGL 321

Sc 162 ----------QKAE**F**KLDCA-SQSKNTIWVHRDECKF HG-EDNKRAFTQQIPLVNIKNNIEIAVNWYNLSGN 221

Pp 202 ----PPQQQKYQCQ**F**KFDVVQ-KKHRYIWVYKEECK- HNFDDKKYAFVQPIQPVVSGDVLKFSLNFYNLNGL 267

Sl 441 ---ESKIEGEYQNV**Y**QFDLYCARANRWLWLHQDECLF HGsTDSR-AYSIPIVPICVEDNIEISLTLISLMGV 508

Ot 559 ------SKQTFYAD**Y**KLDVVKPNSERVVWLHKDEQEQ[14]---DIMNRPYIQPITQICAKDTIEFAINLYCLQGL 635

Sm 800 ----EDRKDPFECS**F**KFDTYTRGRSRNFWIHKDICRF HG-DETRVAATQNITPVCVNDRIEIAVNLFNAMGL 866

Tg 661 VALDKVKWLPARVEWRREAV--- STRGVFNR--- EICPLERCS---PDWLPADQFRIMTTE RLKAPEDF-SP 722

Hh 660 VAVDKVAWLPARVEWRRDAV--- STRGVFNR--- EICPLERCS---PDWLPADQFRIMTTE RLKAPEDF-SP 721

Nc 632 VALDNVHWLPARTEHRQESV--- STRGVFNR--- EICPLERCS---PEWLPADQFRILATE RPKAPEDF-SP 693

Beb 655 VALDRVCWLPPVQERRRDSA--- PSRGIFSR--- EACPLERGA---PDWLPLDQFRVMTTE RLKDADDF-SP 716

Cs 657 VALDRVSWLPLRQEKRRQSL--- PSKGLFSP--- EACPLEWST---AEWFPADEFRLMSTE RVKDPEDF-GP 718

Em 1765 SDVSSIQWFPISIARRVPRV---[9]ASKGLY----- EGCSLELQY---TEWFDGDQYRHMTTE RLQRAECL-EP 1833

Cc 527 ADLHTIRWLPMHVAPRLSRV---[9]AALGLY----- EGCGLEMQY---VEWFDGDQYRHMTTE RLKRAECL-EP 595

To 432 TDVKSIKFLPIVRDVLSKAP--- SHLDLLHR--- EWYTVEPNS----KYLEQLFPQNIHSY SIIHPHKL-LP 492

Bab 397 TNVDSIKFLPMRREAVEGEP---[1]SSLDKMHR--- EWYTVGPNS----HYLQQLFPADIVAY NLVQPEKL-QP 458

Pr 474 VDFNSIKFISNKLSKYIIY---- ----------- NNQLDDQM-----WYNKEEYQILIKEN RLITFESL-LP 524

Cm 389 VNIKSIEFQPISFETIKSE---- ----------- N-CEVEDLYPNTSDWSSIDNQNEVVES --FHIPDF-SP 441

Vb 402 VDMQTFRWMPLRGEPATKQI--- ----------- --FPLEKELD---DWYELDLFQSQTVE RLSVPNHF-EP 453

Im 211 IDLDSITWKEPIFQHIPKIPI--[5]-----------[9]ICEIEQIY---SGWILYNYFHNNQ-- KC-RLDFF-EP 275

Pt 201 IDPDTIEFEEPSIEQSKEC---- ----------- ICPIQSLF---FDWVGIDYAKQKP-- --KLNDYLVHP 249

Tt 322 VNIYSIQWKELTTGSIPSVPI--[5]-----------[9]ICETEEIY---SDWVLYEYFHKIN-- KK-RIDDF-SP 386

Sc 222 IRLDSIQWQTPTIQETKEILKnL[9]PQNDSDGITKK[8]NCELELSQ---TEWYDAKYYLKPSQ- -VYTYDFF-YP 303

Pp 268 VDLSSIYFKSCKISDIPNVPL--[5]-----------[9]ICEIEELY---TDWTLYEYYHRQE-- KI-KLDHF-QP 332

Sl 509 LDTDTVRWQSLKIESQPDQnDIR[9]PNRDLLAKY--[6]VCEVEDTV---VEWFTEPNIKTKSLK[4]RKLDTEQF-SK 592

Ot 636 IDIDSIEWLDCDTTNPPKE-nVI[9]ASSDKAQKI--[6]ICELEDAV---VEWYDSKYFQRQQ-- EMLNIDFL-NE 712

Sm 867 VDVDKVGWRPLEIFPMQDR---- ----------- ECPIEQEL---FDWYDLDSFQSQSVE RLQIPDFF-SP 918

Tg 723 CLKHVKTEFSGM**D**VAVRKSTYRAVRQG-SLGSAACRSWGFPCEI LPQGVPVVCSLTRWGLQHDR-FLSVQL**R**EG**D**I 796

Hh 722 CLKHVKTEFSGM**D**VAVRKSTYRAVRQG-SLGSAACRCWGFPCEI LPQGVPVVCSLTRWGLQHDR-FLSVQL**R**EG**D**I 795

Nc 694 HLKHVKTEFSGM**D**VAVRKSRYRAVRQG-SLGSAACRCWGFPCEI LPQGLPVVCSLTRRGLQHDR-FLSVQL**R**EG**D**V 767

Beb 717 HLRHIHTEFFGM**D**VAVRKSTYRAVRQG-SLGATACRCWGFPCEV LPQGVPIVCPLTRRGLQHDR-FLSVQL**R**EG**D**V 790

Cs 719 YLRHFRTEFSGM**D**VAIRKSTYQAAAPG-SLGVNACRCWGFPCEV LPAGVPIVCPLTRRGLQHDR-FLSVQL**R**VG**D**I 792

Em 1834 ELHHESTEYSGI**D**LLVRRSLYRAMQEG-TAGAAAQRAWGLQCKV VSQEAPIVFPLTRKGLLHDR-STCLQL**R**VG**D**T 1907

Cc 596 ELHHISTSYSGV**D**VLVRRSTYNAAREG-SLGAAAKRAWGLQCRV LPADAPIVFPLTRRGLLHDR-ATSFYL**R**VG**D**T 669

To 493 QLVHQVTQVAGI**D**VITSKSIYVASHTG-MV-VESCNIIGHTVEV VPKTDSIICMIQRIGLQHDR-ICHVQL**R**PG**D**N 565

Bab 459 ELEHQVTQVAGI**D**VITSRSRFIAAKPG-PV-TEAQNFIGHTMEV IDARQPVVCMLQRSGVQCDR-LCHVQL**R**VG**D**K 531

Pr 525 HLKHINTIYSGI**D**VTVMKSTYKAIQPG-KLGKKSYNLWGNYFII EDKNDPVFTFLKREGLQHDYIYHKFYL**R**VG**D**Y 599

Cm 442 NLIITDTEYAGT**D**LVTCRVHYKAVKCG--ELSNAEKIFGIDIVV LPRDYAIICPLKRIGLQHDR-YSPLQI**R**ID**D**T 514

Vb 454 HLRHESTVFSGI**D**IAVSKTRYTAEKPG-RP-EGAERLLGLRLQV VPRDAAVLVPLTRVGLQHDR-FTNLQV**R**PG**D**V 526

Im 276 QLTLKETRCAGV**D**VITSKLIFQAKNPG--VVSKSYENFGINIKV LDNKQEAICQARKLGLFQDL-QNEIQL**R**IG**D**Y 348

Pt 250 HLHLIDCRCAGV**D**TVAYQFVYEACEIG----SFRNELLGIPIEV VQQGQEVVTELNKVGLVSDR-ESKLQL**R**KH**D**Q 320

Tt 387 YLHLKEVYCAGV**D**IITSKHIYIAKQPSNGILQKSKEKLGVYIQI LDDSEDQICEIKRKGLYQDN-ETTIQL**R**VG**D**I 461

Sc 304 FLRLMSSEFAGV**D**VTVSRNTYRAEKIG--IVPDSVARIGILIEI MPKDEEVTYEVKRMGLVYDR-HRPVEL**K**IG**D**T 376

Pp 333 YLKLEEVRCSGV**D**IVTSKHLYKANKVG--QVPKAFENFGVNINI VPSDKEIIAEVKKIGLFHDL-ENTVEL**R**VG**D**F 405

Sl 593 QFDISKVEVGGI**D**FSVWKVQLRAKKNP-LQEYKFYKDLNMGIRI EKDQSLSIINEIKRSGYLIDK-GQDIEL**R**SG**D**F 667

Ot 713 LFVVKKYECAGI**D**GLICKIQLQAVREG----KLSNQYLGISLIV[12]IDQDHGLVNEVKRLGLLIDR-YVDLEL**R**IG**D**T 795

Sm 919 QLMHMSTEYAGI**D**VAVSRTRLRAEAQG--VVPQADRILGSRFEV LPRSSPIILPLKRVGLQHDR-FTKIQL**R**QG**D**C 991

Tg 797 IDYYMSQ**G**GANA* 808

Hh 796 IDYYMSQ**G**GANA* 807

Nc 768 LDYYVSQ**G**GATA* 779

Beb 791 VDYYLSQ**G**GANV* 802

Cs 793 VEFYMSQ**G**GAAV* 804

Em 1908 LESYLTI**G**GANV* 1919

Cc 670 LDSYLTV**G**GGTF* 681

To 566 LQFYITK**G*** 573

Bab 532 VRFYTTK**G*** 539

Pr 600 VVFYLIK**G**GNNI* 611

Cm 515 IVLYITK**G**GAIPE* 527

Vb 527 LYLYISQ**G**GKIIaKR* 541

Im 349 FIFYISK**G**E* 357

Pt 321 LIFYLTS**G**D* 329

Tt 462 FVFYISS**G**D* 470

Sc 377 FVLYISR**G**G* 385

Pp 406 IIVYISK**G**D* 414

Sl 668 LLVYVSM**G**GFEK* 679

Ot 796 LVIYVQR**G**SS* 805

Sm 992 ILLYISQ**G**GKMT* 1003
